# Serum metabolism alteration behind different etiology, diagnosis, and prognosis of disorders of consciousness

**DOI:** 10.1186/s41016-024-00365-4

**Published:** 2024-04-09

**Authors:** Qianqian Ge, Hezhen Lu, Xiaoli Geng, Xueling Chen, Xiaoyan Liu, Haidan Sun, Zhengguang Guo, Jiameng Sun, Feng Qi, Xia Niu, Aiwei Wang, Jianghong He, Wei Sun, Long Xu

**Affiliations:** 1 Department of Neurosurgery, Beijing Tiantan Hospital, Capital Medical University, Beijing, China; 2 China-Japan Union Hospital of Jilin University, Changchun, China; 3 Core Instrument Facility, Institute of Basic Medical Sciences, Chinese Academy of Medical Sciences, School of Basic Medicine, Peking Union Medical College, Beijing, China; 4 China National Clinical Research Center for Neurological Diseases (NCRC-ND), Beijing, China

**Keywords:** Disorders of consciousness, Serum biomarkers, Untargeted metabolomic analysis

## Abstract

**Background:**

Patients with disorders of consciousness (DoC) exhibit varied revival outcomes based on different etiologies and diagnoses, the mechanisms of which remain largely unknown. The fluctuating clinical presentations in DoC pose challenges in accurately assessing consciousness levels and prognoses, often leading to misdiagnoses. There is an urgent need for a deeper understanding of the physiological changes in DoC and the development of objective diagnostic and prognostic biomarkers to improve treatment guidance.

**Methods:**

To explore biomarkers and understand the biological processes, we conducted a comprehensive untargeted metabolomic analysis on serum samples from 48 patients with DoC. Patients were categorized based on etiology (TBI vs. non-TBI), CRS-R scores, and prognosis. Advanced analytical techniques, including PCA and OPLS-DA models, were employed to identify differential metabolites.

**Results:**

Our analysis revealed a distinct separation in metabolomic profiles among the different groups. The primary differential metabolites distinguishing patients with varying etiologies were predominantly phospholipids, with a notable decrease in glycerophospholipids observed in the TBI group. Patients with higher CRS-R scores exhibited a pattern of impaired carbohydrate metabolism coupled with enhanced lipid metabolism. Notably, serum concentrations of both LysoPE and PE were reduced in patients with improved outcomes, suggesting their potential as prognostic biomarkers.

**Conclusions:**

Our study underscores the critical role of phospholipid metabolism in the brain’s metabolic alterations in patients with DoC. It identifies key biomarkers for diagnosis and prognosis, offering insights that could lead to novel therapeutic targets. These findings highlight the value of metabolomic profiling in understanding and potentially treating DoC.

**Supplementary Information:**

The online version contains supplementary material available at 10.1186/s41016-024-00365-4.

## Background

Following severe brain injury, such as from a traumatic event, hypoxic-ischemic encephalopathy after cardiac arrest, or a massive stroke, a disorder of consciousness (DoC) may ensue [1]. The acute and short-term DoC is known as coma, while the prolonged DoC is further divided into vegetative state (VS) or minimally conscious state (MCS) by the state of wakefulness and awareness of patients [2]. Recent advancements in treating cerebral injuries have reduced the mortality rate of severe brain injuries [3]. However, patients and their families aspire for more than mere survival and awakening; they yearn for interaction, communication, and a return to living. This aspiration is echoed by medical professionals globally, underscoring the importance of treating DoC as critically as the primary disease.

The Coma Recovery Scale-Revised (CRS-R) is the most widely utilized tool for measuring levels of consciousness, evaluating a range of behavioral responses to distinguish cognitively mediated behavior from reflexive activity [4]. Diagnosis [5], prognosis [6], and assessment of treatment efficacy [7] in DoC are heavily dependent on CRS-R scores. However, the behavioral markers on which CRS-R relies are variable, and clinician assessments can be subjective, leading to a high rate of misdiagnosis [8]. Additional neuroimaging techniques, such as structural and functional MRI, EEG-evoked potentials, and near-infrared spectroscopy, are employed to assess the consciousness state and treatment response in DoC patients [9]. Despite significant advancements in neuroimaging, these techniques are not easily accessible or suitable for continuous monitoring. There is a need for a laboratory diagnostic biomarker for DoC, akin to the role of brain natriuretic peptide in diagnosing and monitoring congestive heart failure, to be established.

Metabolomics, a burgeoning omics approach, enables the unbiased identification and quantification of thousands of small-molecule metabolites in biofluids [10]. Both physiological and pathological processes are reflected in cellular metabolism, leading to changes in the chemical composition of interstitial fluid. Given that cerebrospinal fluid, the brain’s waste removal system, is challenging to collect and is absorbed into the bloodstream [11], blood serum presents a more practical sample for studying metabolic changes in the central nervous system. Utilizing this profiling technology, alterations in numerous biomarkers have been identified in central nervous system disorders, such as traumatic brain injury (TBI) [12], ischemic stroke [13, 14], and hypoxic-ischemic encephalopathy [15], which are common causes of DoC. To date, only one targeted metabolomics study has explored this uncharted territory. Yu, J. et al. [16] investigated 12 VS patients, 11 MCS patients, and 8 healthy controls for targeted metabolomic analysis, and 6 increased and 2 decreased metabolites were identified in MCS and VS groups compared to healthy controls. However, none of these metabolites showed a significant difference between these two consciousness states.

Beyond identifying metabolic changes between healthy individuals and DoC patients, our study aims to understand the biological processes associated with varying levels of consciousness and the factors enabling some patients to recover from DoC. We conducted untargeted metabolomic analysis on 48 DoC patients, categorizing them into two groups based on their pathogenesis, CRS-R scores, and prognosis, and conducted analyses across these three dimensions. Our objectives are to (i) explore whether DoC arising from different pathogeneses exhibit distinct pathological processes; (ii) pinpoint blood metabolites that mirror levels of consciousness; and (iii) identify biomarkers predictive of recovery from DoC. The outcomes of this study promise to offer novel insights into the diagnosis and prognosis of DoC and potentially illuminate the role of metabolic disturbances in its pathogenesis.

## Methods

### Patient enrollment and assessment

For our prospective cohort study, patients were recruited from March to September 2021 at Beijing Tiantan Hospital. The study included patients admitted for treatment of disorders of consciousness (DoC). The inclusion criteria were as follows: diagnosis of DoC based on the Coma Recovery Scale-Revised (CRS-R), condition persisting for over a month, sudden onset of DoC, and informed consent obtained from family members. Exclusion criteria were: neurodegenerative diseases, intracranial infections, post-surgical coma due to brain tumors; patients whose consciousness level continuously improved or deteriorated in the month before enrollment; patients with recurrent, difficult-to-control epileptic seizures; those with severe complications or any signs of acute infection; patients who had participated in other clinical trials within 3 months prior to this study; and patients with a prognosis of limited life expectancy. Each patient underwent either cranial CT scans or MRI to confirm the causes of their DoC. Follow-up assessments using the CRS-R were conducted 3 months post-treatment to gauge prognosis. The patients were categorized based on etiology (traumatic vs. non-traumatic brain injury), diagnostic state (CRS-R ≤ 7 vs. CRS-R > 7), and prognostic outcomes (improvement or no improvement in CRS-R score after 3 months). This investigation adhered to the principles of the Declaration of Helsinki. The study protocol received ethical clearance from the Ethics Committee of Beijing Tiantan Hospital (Approval No. KYSQ 2021–396-01).

### Sample collection and processing

For each participant, a 200-μL blood sample was collected before treatment. To this, an equal volume of acetonitrile (200 μL) was added, initiating protein precipitation. The mixture was then subjected to vigorous vortexing for 30 s, followed by centrifugation at 14,000 × g for 10 min. The resulting supernatant was carefully extracted and dried under a vacuum. The dried extracts were reconstituted in 200 μL of 2% acetonitrile solution for further analysis. Prior to LC-MS analysis, metabolites were isolated from larger biomolecules using ultra-centrifugation filters with a 10 kDa molecular weight cut-off (Millipore Amicon Ultra, MA). The prepared samples were then methodically transferred to autosamplers for subsequent metabolomic profiling.

### Quality control measures

To ensure analytical consistency and reliability, a quality control (QC) protocol was rigorously followed. The QC samples were created by pooling aliquots from all the samples across patient groups. These QC samples were interspersed randomly throughout the analytical sequence. This strategy was employed to monitor the stability and repeatability of the LC-MS system, providing a robust set of reference data against which sample readings could be compared and validated.

### LC-MS analysis

The metabolomic profiling of the samples was performed using an advanced ultra-performance liquid chromatography-mass spectrometry (LC-MS) setup. This involved the integration of a Waters ACQUITY H-class LC system with a TripleTOF 5600 mass spectrometer (AB SCIEX, MA, USA). Chromatographic separation of metabolites was achieved using a Waters HSS C18 column (dimensions: 3.0 × 100 mm, particle size: 1.7 μm). The flow rate was maintained at 0.3 ml/min throughout the process.

The mobile phases used were as follows: Phase A, consisting of 0.1% formic acid in water, and Phase B, comprising acetonitrile. The gradient profile was meticulously programmed as follows: initial 2% B for the first 2 min, a gradual increase from 2% to 55% B over the next 3 min, a steep rise to 100% B from 5 to 15 min, a hold at 100% B until 20 min, a swift change back to 2% B at 20.1 min, and a final equilibration at 2% B for the remaining 8.9 min. The column was thermostatically controlled at 50°C.

For mass spectrometric analysis, the samples underwent a full scan from 50 to 1200 m/z. The parameters set for the mass spectrometer included a full-scan accumulation time of 0.25 s, an MS/MS accumulation time of 0.1 s, GAS1 and GAS2 at 55, a source temperature of 550°C, an ionization spray voltage of 4500V, and a collision energy of 35 for MS/MS scans. The top 100 precursors identified in the full scan were selected for MS/MS analysis, with a dynamic exclusion duration of 5 s.

### Data processing and analysis

We processed the LC-MS raw data using Progenesis QI software (Waters, Milford, MA, USA), focusing on normalization through missing value estimation, log transformation, and Pareto scaling, facilitated by Metaboanalyst 5.0 (http://www.metaboanalyst.ca). Variables missing in over 50% of samples were excluded to maintain data quality.

Statistical significance was assessed using non-parametric Wilcoxon rank-sum tests, with false discovery rate (FDR) correction applied for multiple hypothesis testing. Pattern recognition involved principal component analysis (PCA) and orthogonal partial least squares discriminant analysis (OPLS-DA) using SIMCA 14.0 (Umetrics, Sweden). Criteria for significant variables included a fold change (FC) ≥ 1.5 and a variable importance in the projection (VIP) value > 1.0 from OPLS-DA. Further analysis, including ROC analysis and biomarker validation, was conducted using the “Biomarker discovery” module on Metaboanalyst 5.0, focusing on identifying diagnostic and prognostic relevant biomarkers.

## Results

### Subjects

**Fig. 1:**
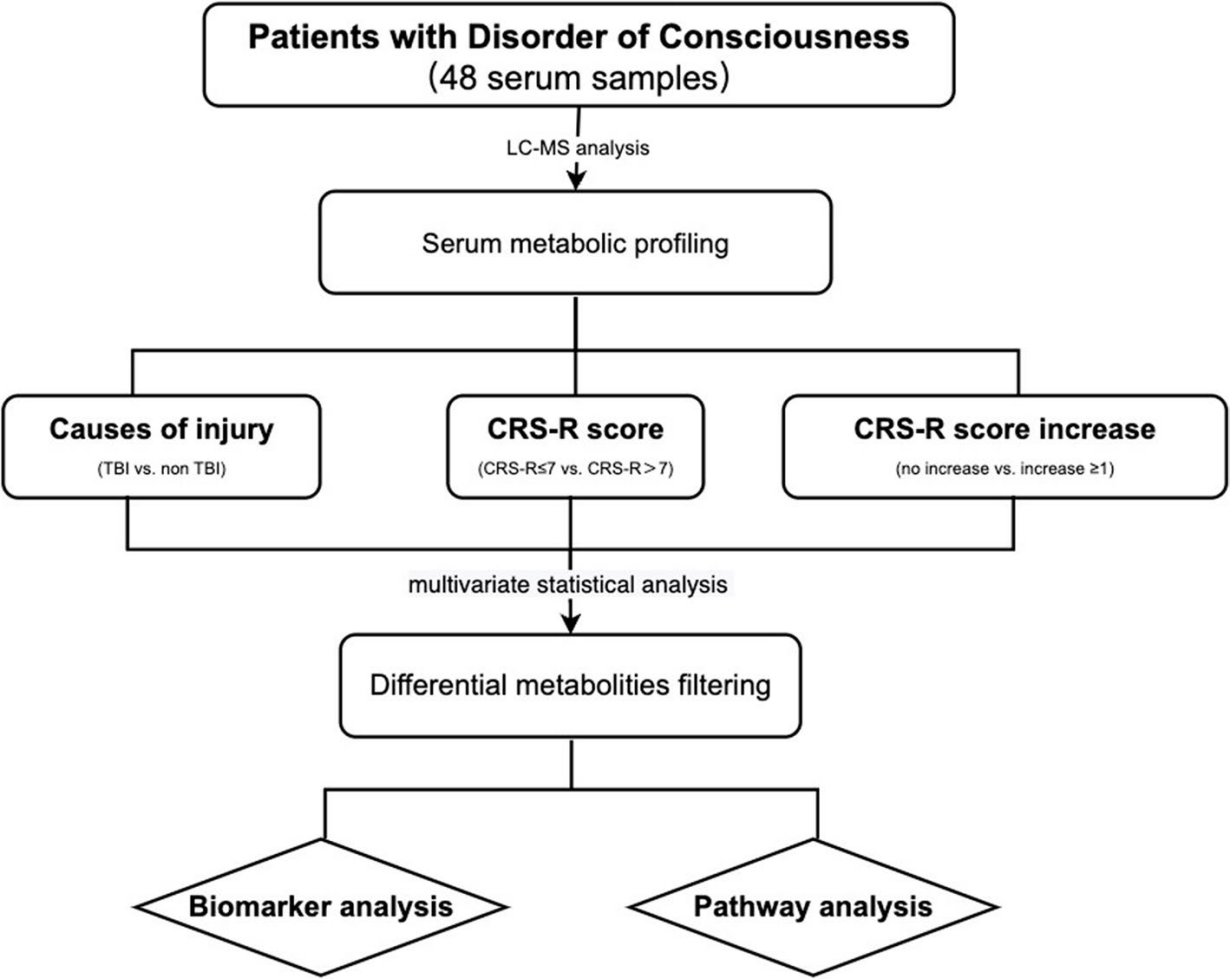
Study design of the serum metabolomic study in DoC patients. CRS-R, Coma Recovery Scale-Revised; TBI, traumatic brain injury

Our objective was to explore the pathological processes underlying DoC resulting from various brain injuries, identify blood metabolites indicative of consciousness levels, and discover serum biomarkers predictive of patient recovery rates. We recruited 48 patients experiencing DoC for over a month and collected their blood samples. Figure 1 illustrates the workflow of our study, while Table 1 presents the baseline clinical data of all participants. We observed no significant differences in clinical and demographic characteristics across the groups, except for the criteria used for grouping. The blood samples were centrifuged, and only the plasma was retained for subsequent LC-MS analysis. To evaluate data quality, we calculated the coefficient of variation in metabolite abundance across six replicates of the QC sample. The Pearson correlation coefficients in all replicates exceeded 0.8, demonstrating robust technical reproducibility (Additional Fig. 1).

**Table 1:** Clinical characteristics of patients with DoC

	Etiology		CRS-R		Increase	
Characteristics	Non-TBI	TBI	≤ 7	> 7	0	≥ 1
Patients (*n*)	26	22	32	16	30	18
Male/female (*n*)	16/10	16/6	21/11	11/5	20/10	12/6
Age (years)	51.8 ± 13.0	49.3 ± 17.6	50.6 ± 15.5	50.8 ± 14.8	50.0 ± 12.4	51.8 ± 19.1
Duration (months)	5.2 ± 4.1	8.0 ± 7.4	6.2 ± 4.7	7.2 ± 7.9	6.8 ± 6.9	6.0 ± 4.1
TBI/nonTBI (*n*)	0/26	22/22	15/17	7/9	14/16	8/10
CRS-R	7.0 ± 3.7	6.6 ± 3.1	4.7 ± 1.4	11.1 ± 2.2	6.4 ± 3.6	7.5 ± 3.0
Increase	0.5 ± 0.7	0.7 ± 1.1	0.7 ± 1.0	0.4 ± 0.7	0	1.6 ± 0.8

Continuous variables are expressed as mean ± standard deviation (SD), *TBI* traumatic brain injury, *CRS-R* Coma Recovery Scale-Revised score

### Metabolic difference between different pathogenesis of DoC

**Fig. 2:**
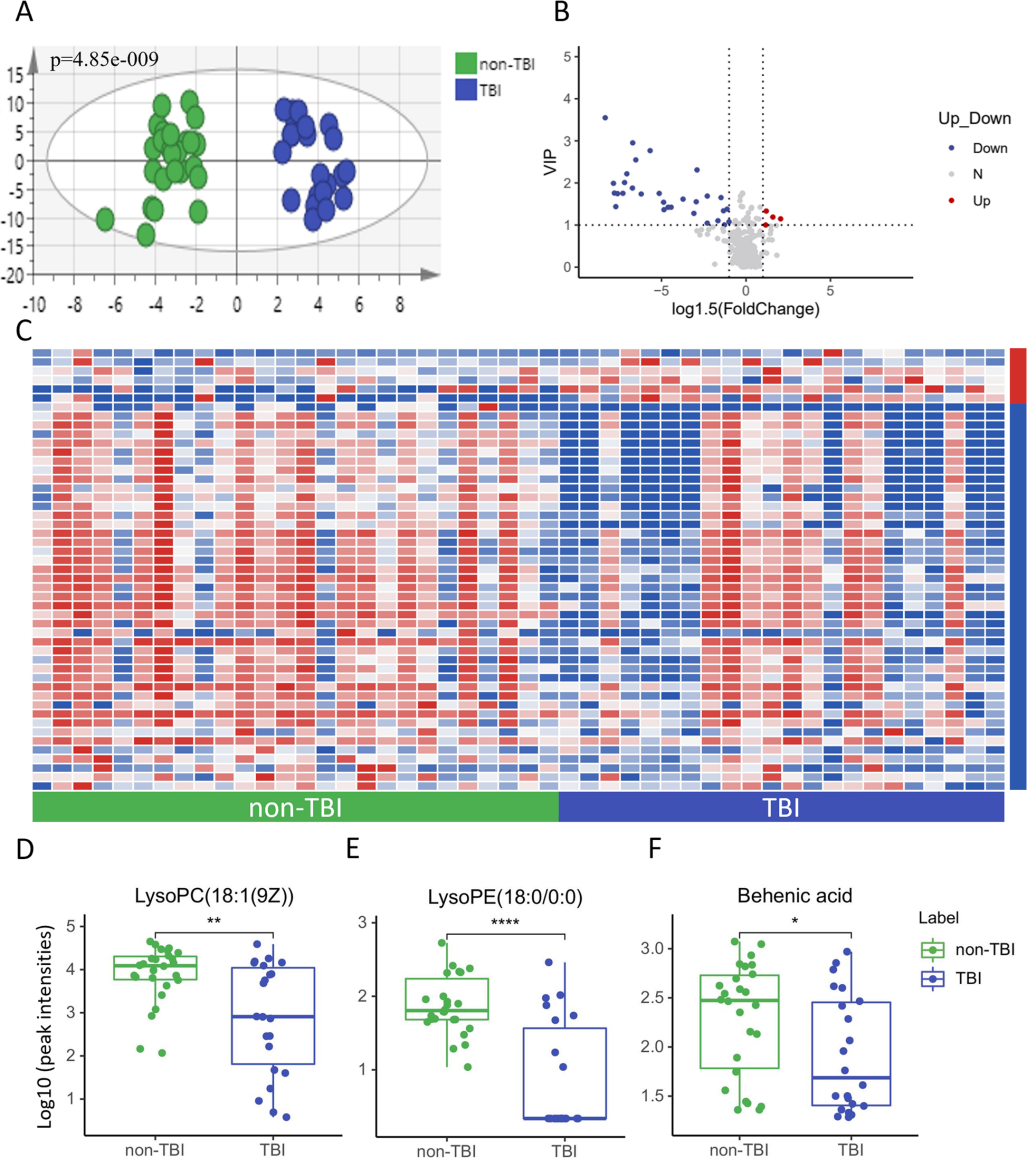
Analysis of serum metabolomics of TBI-induced DoC and non-TBI-induced DoC. **A** OPLS-DA model based on patients’ serum metabolites for classification of TBI-induced and non-TBI-induced DoC; **B** volcano plot for filtering differential metabolites (VIP value ≥ 1, fold change ≥ 1.5); **C** heatmap of differential metabolites in the two groups (blue indicate relative lower and red indicate relative higher in TBI-induced DoC group); **D**–**F** box plots for relative intensities of lysoPC(18:1(9Z)), lysoPE(18:0/0:0), and behenic acid in non-TBI-induced and TBI-induced DoC

We utilized unsupervised PCA (Additional Fig. 2A) and a supervised OPLS-DA model to identify the preliminary difference in the metabolic profile of DoC caused by different brain-damage events. The score plots of OPLS-DA (Fig. 2A) showed a significant difference between the two groups. The volcano plot (Fig. 2B) was constructed based on the variable importance in the projection (VIP) from the OPLS-DA model (Additional Fig. 2C) and the logarithm to base 1.5 of the FoldChange. Forty-nine differential metabolites between the TBI and non-TBI groups were selected based on VIP ≥ 1 and FoldChange ≥ 1.5, as shown in a heatmap (Fig. 2C). Further analysis of these metabolites revealed that 73.5% are lipids and lipid-like molecules, with a notable decrease in 24 glycerophospholipids in the TBI group. The relative content of representative metabolites in these altered metabolic pathways is presented in box diagrams. LysoPC(18:1(9Z)) (Fig. 2D), lysoPE(18:0/0:0) (Fig. 2E), and behenic acid (Fig. 2F) were significantly lower in the TBI group. To further assess the discriminative capacity of these metabolites, receiver operator characteristic (ROC) curves were applied, and the areas under the ROC curve (AUC) are listed in Additional Table S1. According to the ROC curves, 19 metabolites demonstrated good performance in distinguishing TBI-induced DoC from non-TBI cases, with AUC values above 0.7. Five of these, N-furfurylformamide, desacetycefapirin, lysoPE(18:0/0:0), polyoxyethylene 40 monostearate, and lysoSM(d18:1), showed even better discriminative ability with AUC values above 0.8. Additional statistical data for the top ten endogenous metabolites in AUC are provided in Table 2.

**Table 2:** Biomarkers between different pathogeneses of DoC

Biomarkers	AUC	VIP	FC
N-Furfurylformamide	0.94056	7.45413	3.72E-06
LysoPE(18:0/0:0)	0.83392	3.54948	0.034073
Polyoxyethylene 40 monostearate	0.80944	3.98297	0.013319
LysoSM(d18:1)	0.80245	3.60472	0.012899
LysoPC(20:2(11Z,14Z))	0.78147	3.51983	0.008518
Met Phe Thr Glu Asp	0.75874	2.54835	0.071025
PC(17:1(9Z)/0:0)	0.75175	3.41342	0.011463
PE(18:1(9Z)/0:0)	0.74126	2.85349	0.00396
LysoPC(18:1(9Z))	0.73601	2.95301	0.065963
LysoPE(22:5(4Z,7Z,10Z,13Z,16Z)/0:0)	0.73601	3.20381	0.019468

*AUC* area under curve, *VIP* Variable Importance in Projection, *FC* fold change

### Metabolic difference between different CRS-R scores

**Fig. 3:**
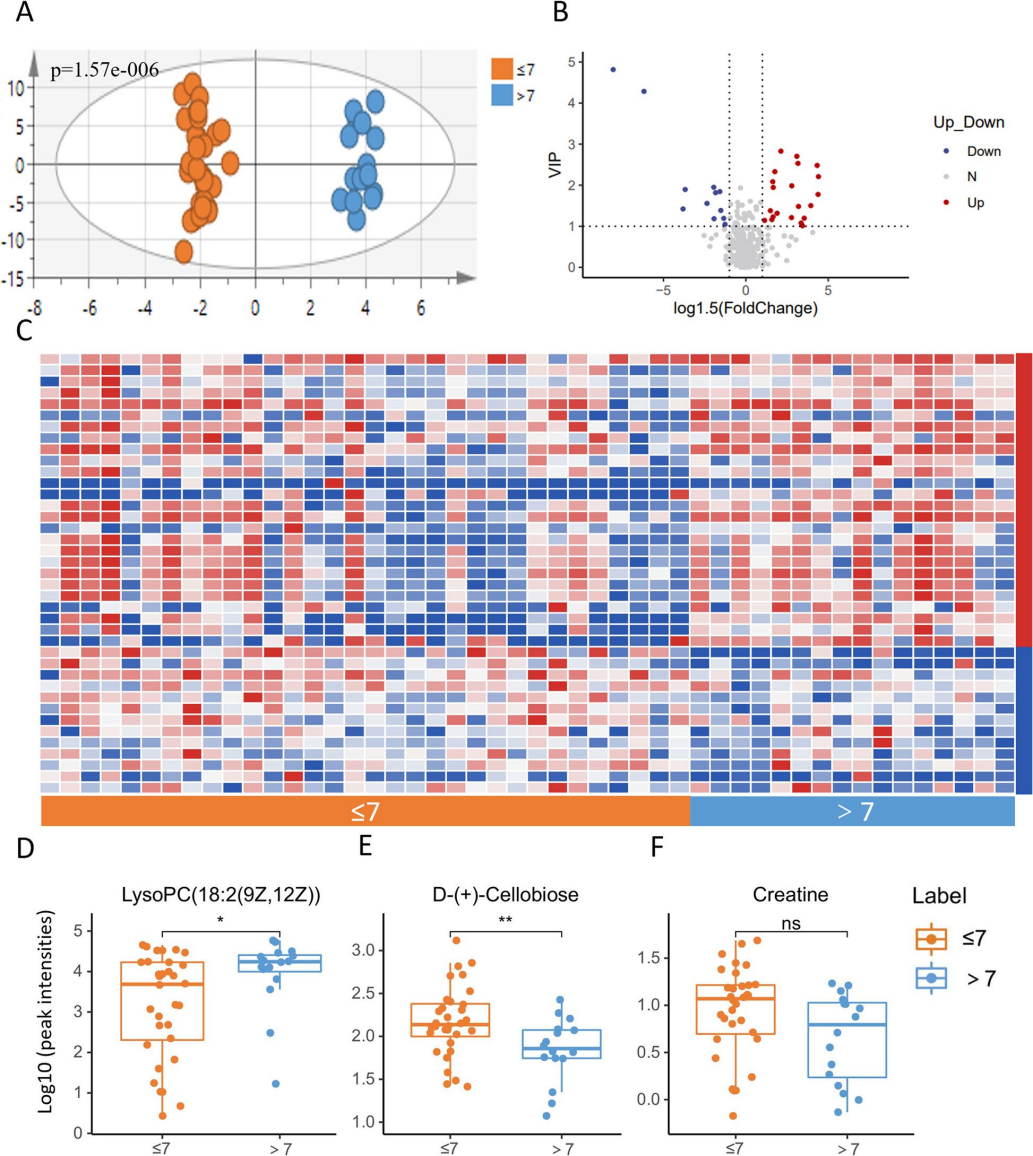
Analysis of serum metabolomics of DoC patients with different CRS-R scores. **A** OPLS-DA model based on patients’ serum metabolites for classification of patients with CRS-R scores less than or equal to 7 and patients with CRS-R scores greater than 7; **B** volcano plot for filtering differential metabolites (VIP value ≥ 1, fold change ≥ 1.5); **C** heatmap of differential metabolites in the two groups (blue indicate relative lower and red indicate relative higher in the group with higher CRS-R scores); **D**–**F** box plots for relative intensities of lysoPC(18:2(9Z,12Z)), D-(+)-cellobiose, and creatine in DoC patients with different CRS-R scores

Similarly, we analyzed the overall differences between two groups categorized by different CRS-R scores using PCA (Additional Fig. 3A) and OPLS-DA (Fig. 3A) models. The OPLS-DA model’s score plot revealed a clear separation between the groups. The distribution of all detected metabolites is depicted in the volcano plot (Fig. 3B), utilizing VIP values (Additional Fig. 3C) and log1.5 (FoldChange) as coordinates. The two groups, differentiated by CRS-R scores, exhibited a total of 39 differential metabolites, adhering to the criteria of VIP ≥ 1 and FoldChange ≥ 1.5. Of these, 26 metabolites increased and 13 decreased in the group with higher CRS-R scores, as illustrated in the heatmap (Fig. 3C). Lipids and lipid-like molecules constituted 46.2% of the differential metabolites, with 72.2% being glycerophospholipids, all of which increased in the group with higher CRS-R scores. Notably, lysoPC(18:2(9Z,12Z)) (Fig. 3D) increased significantly, while D-(+)-cellobiose (Fig. 3E) and creatine (Fig. 3F) decreased in the group with higher scores. The discriminative capacity of each selected differential metabolite was further assessed through ROC curve analysis (Additional Table S2). The analysis revealed that 14 metabolites had strong diagnostic value for differentiating CRS-R scores, with AUC values above 0.7, and 5 of these exhibited even greater separating capacities with AUC values above 0.8. Additional statistical data for these metabolites are presented in Table 3, excluding 4 exogenous metabolites (Table 3).

**Table 3:** Biomarkers between DoC patients with different CRS-R scores

Biomarkers	AUC	VIP	FC
1α-hydroxy-25,26,27-trinorvitamin D3 24-carboxylic acid	0.86328	4.81561	0.03815
N-Furfurylformamide	0.81055	6.06563	53572.37
LysoSM(d18:1)	0.78906	3.81306	65.23973
LysoPE(18:0/0:0)	0.78125	3.27009	41.44823
3,4,5-trihydroxy-6-{3-[2-(3-hydroxy-5-methoxyphenyl)ethyl]phenoxy}oxane-2-carboxylic acid	0.75879	1.77463	5.919864
D-(+)-Cellobiose	0.73242	1.84418	0.526984
PE(18:1(9Z)/0:0)	0.7168	2.7041	3.508338
2-[(6-carboxy-3,4,5-trihydroxyoxan-2-yl)oxy]-3-hydroxybutanedioic acid	0.71484	1.81671	0.475243
7-Hydroxyoctanoic acid	0.71289	2.48143	5.784867
Molybdopterin precursor Z	0.70898	1.14197	1.586331

*AUC* area under curve, *VIP* Variable Importance in Projection, *FC* fold change

### Metabolic difference between different prognoses of DoC

**Fig. 4:**
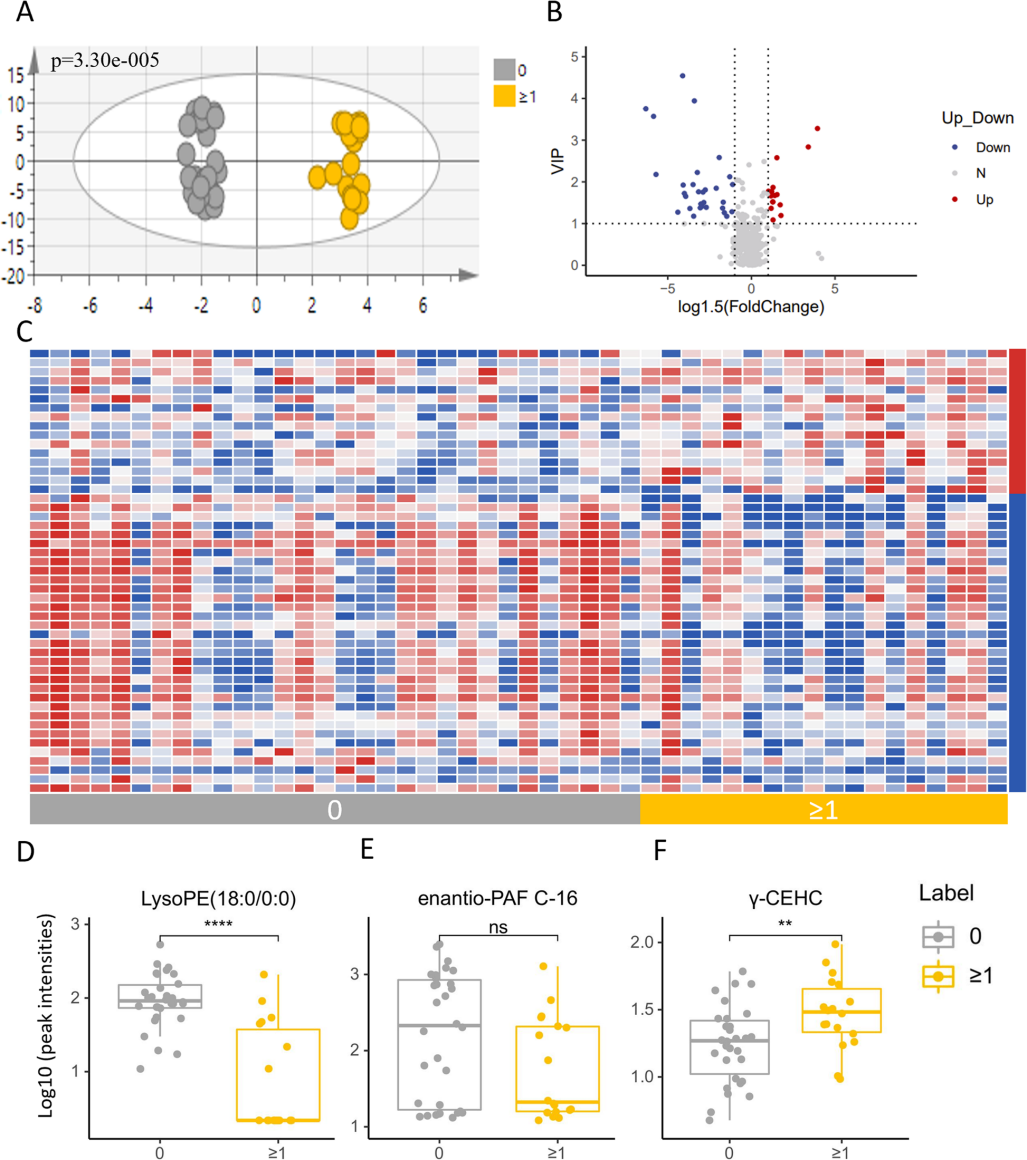
Analysis of serum metabolomic of DoC patients with different prognoses. **A** OPLS-DA model based on patients’ serum metabolites for classification of patients with and without improvement after 3 months; **B** volcano plot for filtering differential metabolites (VIP value ≥ 1, fold change ≥ 1.5); **C** heatmap of differential metabolites in the two groups (blue indicate relative lower and red indicate relative higher in the group with higher CRS-R scores); **D**–**F** box plots for relative intensities of lysoPE(18:0/0:0), enantio-PAF C-16, and y-CEHCin DoC patients with different prognosis

We conducted analyses to identify metabolites that could distinguish patients with the potential to regain consciousness. PCA did not reveal clear discrimination between the two groups with different outcomes (Additional Fig. 4A). However, an OPLS-DA model demonstrated significant separation (*p* < 0.01) (Fig. 4A), with 88 features (Additional Fig. 4C) contributing to the distinction between groups (VIP ≥ 1). Of these, 49 metabolites with a fold change greater than 1.5 were selected as differential metabolites for further investigation. A heatmap of these metabolites (Fig. 4C) showed that 16 were up-regulated and 33 were down-regulated in patients with improved performance. Notably, 20 of the differential metabolites were phospholipids, including 12 phosphatidylcholines (PC) and 8 phosphatidylethanolamines (PE), all of which were present in lower concentrations in the group with increased scores. The relative content of representative metabolites, such as lysoPE(18:0/0:0) (Fig. 4D), enantio-PAF C-16 (Fig. 4E), and phenylacetylglutamine (Fig. 4F), is depicted in Fig. 4. ROC analysis (Additional Table S3) indicated that 19 of these metabolites have significant diagnostic value for predicting the prognosis of DoC, with AUC values above 0.7 (Table 4). Metabolites like lysoPE(18:0/0:0), lysoPE(22:5(4Z,7Z,10Z,13Z,16Z)/0:0), Met Phe Thr Glu Asp, and 6-hydroxyindolelactate demonstrated superior separating capacity with AUC values above 0.8, suggesting their potential as reliable biomarkers for DoC prognosis.

**Table 4:** Biomarkers between different prognoses of DoC

Biomarkers	AUC	VIP	FC
LysoPE(18:0/0:0)	0.8963	4.95737	0.023807
LysoPE(22:5(4Z,7Z,10Z,13Z,16Z)/0:0)	0.87222	2.58399	0.458074
Met Phe Thr Glu Asp	0.87037	3.94251	0.250696
6-Hydroxyindolelactate	0.82037	4.83397	0.009005
LysoSM(d18:1)	0.79815	4.88562	0.018151
PC(17:1(9Z)/0:0)	0.78704	4.54307	0.188814
1α-hydroxy-25,26,27-trinorvitamin D3 24-carboxylic acid	0.77778	2.83801	3.973629
1-Ipomeanol	0.76852	1.86707	1.687214
LysoPC(17:0)	0.7463	3.57104	0.093256
γ-CEHC	0.73333	1.76604	1.636749

*AUC* area under curve, *VIP* Variable Importance in Projection, *FC* fold change

## Discussion

Our LC-MS-based metabolomic approach revealed distinct serum metabolic profiles between DoC patients triggered by TBI and those resulting from other types of brain damage. The metabolomic data, categorized by different CRS-R scores, demonstrated effective separation in the OPLS-DA model, exhibiting high predictability in both relative integral and whole spectra data. Furthermore, the serum metabolomic profile at admission was able to differentiate patients who showed an increase in CRS-R scores at discharge from others. The discriminative potential of specific differential metabolites between each group was also validated, as evidenced by high AUCs. These findings not only underscore the potential application value of metabolomics in the medical decision-making for patients with DoC but may also enhance our understanding of the pathogenic mechanisms underlying disorders of consciousness. To the best of our knowledge, this is the first study to explore metabolomic studies in relation to different pathogeneses and prognoses of DoC.

Traumatic brain injury is one of the leading causes of DoC. The recovery rate of DoC varies with the pathogenesis [17, 18], and TBI-induced DoC patients are more likely to regain consciousness [17]. Among patients in a vegetative state at an early phase of TBI (2 weeks), 78% [19] regained consciousness by 12 months. Unlike hypoxia from cerebrovascular diseases, external force in TBI directly damages neurons and axons, suggesting a distinct pathological process from other DoC causes. Our detection of a unique metabolic profile between TBI and non-TBI groups supports this hypothesis. Of the 49 different metabolites identified between these groups, 24 were phospholipids, and their abundance in the TBI group was consistently lower. A previous serum metabolomics study on acute TBI linked phospholipids closely with TBI severity and as strong predictors of patient outcomes [20]. Brain tissue is phospholipid-rich, predominantly phosphatidylethanolamine (32%) and phosphatidylcholine (26%) [21]. Phosphatidylcholine (PC), a choline-containing phospholipid, crosses the blood-brain barrier via LDL-receptor-facilitated transcytosis [22]. Central choline levels reflect membrane breakdown in damaged cells [23], and circulating PC levels correlate with favorable outcomes [20]. Our data indicate lower serum PC levels in TBI-induced DoC compared to other causes, suggesting more severe symptoms in mild brain damage cases. Phosphatidylethanolamine (PE), also known as cephalins, is primarily found in brain tissue. PE peroxidation is linked to ferroptosis, a regulated cell death form triggered by TBI, leading to neuronal death [24]. However, the studies on the changes in PE after TBI have shown inconsistent results [25–28], mostly focusing on brain tissue levels and acute time points. Our findings of lower serum PE levels in TBI cases after one month compared to other brain injuries warrant further investigation. Four fatty acyls, including behenic acid, showed differential expression between TBI and non-TBI groups, indicating varied energy metabolism patterns. To our knowledge, this is the first study to examine metabolic differences between TBI and non-TBI-induced DoC, and the complex metabolic variations we uncovered require further validation.

We also analyzed the metabolism difference between patients with different CRS-R scores. A similar study by Jie Yu et al. [16] identified PC (38:5) and arachidonic acid as serum biomarkers to distinguish VS patients from MCS patients. However, due to limited sample size, no statistically significant differences in metabolites other than lipids were found between the MCS and VS groups. We enrolled more DoC patients and conducted untargeted metabolomic analysis to verify and expand upon this study. The lipid metabolism level in patients with higher CRS-R scores and better performance was generally elevated, aligning with previous studies that suggest better energy availability for conscious activity [29]. Except for lipid metabolism, we observed lower serum levels of three carbohydrates, including D-(+)-cellobiose, maltotriose, and 2-[(6-carboxy-3,4,5-trihydroxyoxan-2-yl)oxy]-3-hydroxybutanedioic acid, in the higher score group. The main energy source of the brain is glucose, derived from carbohydrates. The down-regulation of carbohydrate metabolism and enhanced lipid metabolism in patients with higher CRS-R scores may indicate a shift in energy metabolism towards a keto-dependent pattern. The beneficial effects of ketone bodies on brain metabolism in various neurological disorders have been widely studied [29], yet their role in DoC remains unclear and warrants further research. Our data also revealed an interesting correlation between DoC and creatine, a growing field in brain research [30]. Creatine facilitates rapid energy provision by transferring the N-phosphoryl group and resynthesizing adenosine triphosphate, crucial for maintaining adenosine triphosphate homeostasis and low ADP concentrations during high energy turnover [31]. It may also attenuate reactive oxygen species formation, suggesting potential therapeutic effects in neurodegenerative diseases [32]. Under certain pathological conditions, reducing creatine kinase activity in the striatum can lead to neurological impairments and basal ganglia abnormalities [33], potentially accompanied by a relative increase in creatine levels. Evidence suggests that creatine supplementation is most effective under cognitive stress, such as during complex tasks, experimental hypoxia, or sleep deprivation [34]. Our data showed relatively low creatine levels in patients with better conscious states, possibly due to higher energy consumption utilizing more creatine. Along with the observed changes in lipid and carbohydrate metabolism, we hypothesize that DoC induces broad alterations in energy metabolism. Understanding this new energy metabolism pattern in DoC could aid in discovering novel treatments, such as ketogenic diets or creatine supplements, representing an exciting area for future research.

Another significant aspect of our research is the identification of serum biomarkers capable of distinguishing patients with the potential for recovery. Predicting recovery from DoC is sometimes more critical than treatment itself, as it influences decisions regarding the continuation of life-sustaining treatments [35]. However, current clinical approaches to this complex issue are often subjective or inconvenient, leading to inconsistent and error-prone decision-making by clinicians. Therefore, the objective and easily accessible serum biomarkers of prognosis identified in our study have the potential to enhance the clinical decision-making process for DoC. Among all the differential metabolites we detected, LysoPE (18:0/0:0) demonstrated the highest AUC value (0.8963), with a sensitivity of 83.3% and specificity of 86.7% for predicting improvement. LysoPE has been reported to predict postischemic cognitive impairment in rats [36]. The endogenous lysophospholipid metabolism pathway translocates dietary LysoPE to plasma, where it is acylated to form PE [37]. Typically, PE and LysoPE levels change in opposite directions. Intriguingly, our results show that serum concentrations of both LysoPE and PE were lower in patients showing improvement, possibly indicating a high demand for PE during neural network reconstruction. Thus, PE supplementation may emerge as a new treatment target for DoC. Another differential metabolite, enantio-PAF (C-16), decreased in the group with increased CRS-R scores. Enantio-PAF (C-16) is a bioactive phospholipid involved in various cellular responses. PAF can induce platelet aggregation and neutrophil release, leading to the production of reactive oxygen species and leukotrienes [38]. In neurodegenerative diseases, the excessive presence of PAF or the decrease in lipid levels is associated with the inflammatory process [39]. PAF’s cytotoxic effects on neuronal cells, including blood-brain barrier disruption and vasoconstriction [40], are mitigated by PAF antagonists [41]. C-16 as an enantiomer of PAF has a similar but weaker effect to PAF [42, 43]. However, the role of C-16 in brain damage and repair is understudied and requires further investigation. We also observed higher serum levels of γ-CEHC in patients with better prognoses. Gamma-carboxyethyl hydroxychroman (γ-CEHC), a metabolite of Vitamin E, reflects Vitamin E metabolism [44]. Tocotrienols and tocopherols, Vitamin E family members, are potent antioxidants protecting cell membranes from oxidative damage. Tocotrienol supplementation has been shown to protect against stroke-induced neurodegeneration [45], and improve motor deficits and neuronal functions in Parkinson’s disease [46]. The increased γ-CEHC levels in patients with better prognoses might indicate higher Vitamin E consumption during brain function reconstruction. However, these findings are preliminary, and further research is needed to validate the prognostic and therapeutic potential of these differential metabolites.

However, this study is subject to several limitations. Firstly, the potential influence of varied therapies received by patients, considering their complex complications, might not have been entirely accounted for. Additionally, we did not account for the potential impact of varying diets. Secondly, the sample size in our study is relatively small, and we did not include healthy controls for comparison. Lastly, the prognostic accuracy of the biomarkers we identified requires external validation and verification through other methods, including but not limited to in vitro and in vivo experiments.

## Conclusions

In conclusion, our study presents a pioneering exploration into the serum metabolomic profiling of disorders of consciousness, particularly focusing on differences arising from TBI versus non-TBI causes. Our findings reveal distinct metabolic signatures correlating with different etiologies, consciousness levels as measured by CRS-R scores, and potential for recovery. Key biomarkers, particularly in lipid and carbohydrate metabolism, have been identified, offering new insights into the underlying pathophysiological mechanisms of DoC. Moreover, we identified 19 differential metabolites as biomarkers that have a good ability to predict the recovery of consciousness. These findings pave the way for future research, which could lead to more effective diagnostic and therapeutic strategies for DoC.

### Supplementary Information

**Additional file 1:** **Figure 1.**

**Additional file 2: Figure 2.**

**Additional file 3:** **Figure 3.**

**Additional file 4:** **Figure 4.**

**Additional file 5:** **Table 1.**

**Additional file 6:** **Table 2.**

**Additional file 7:** **Table 3.**

## Data Availability

The data that support the findings of this study are available on request from the corresponding author. The data are not publicly available due to privacy or ethical restrictions.

## Abbreviations

DoC: Disorders of consciousness
MCS: Minimally conscious state
VS: Vegetative state
CRS-R: Coma Recovery Scale-Revised
TBI: Traumatic brain injury
VIP: Variable importance in the projection
PC: Phosphatidylcholine
PE: Phosphatidylethanolamine
PAF: Platelet-activating factor
γ-CEHC: Gamma-carboxyethyl hydroxychroman
