# Supplementary figures and images for: Serum metabolism alteration behind different etiology, diagnosis, and prognosis of disorders of consciousness
Source: Chin Neurosurg J. 2024 Apr 9;10:12. doi: 10.1186/s41016-024-00365-4 (PMC11003070; doi:10.1186/s41016-024-00365-4)

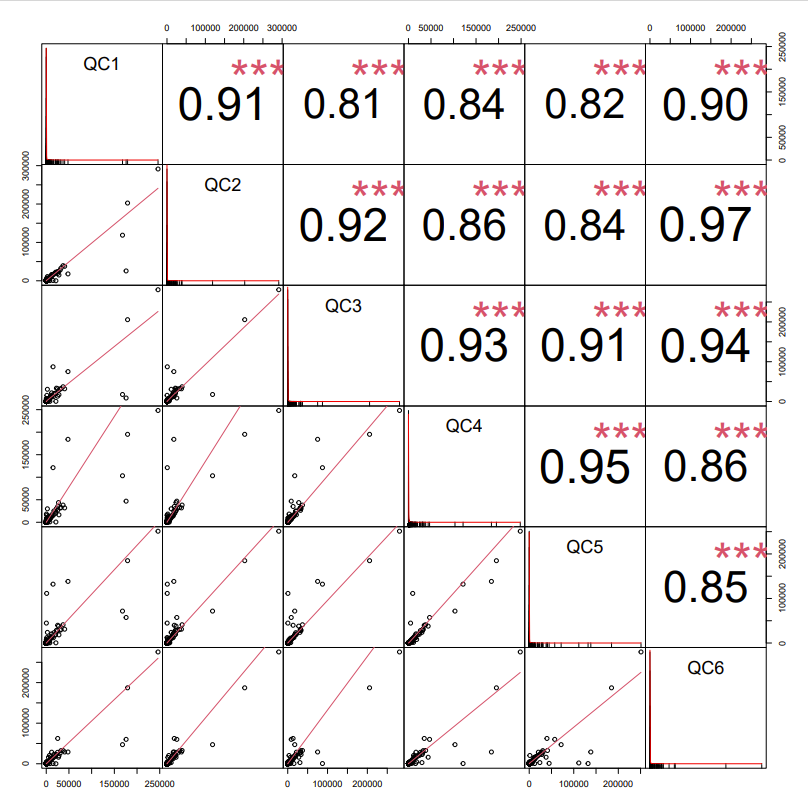

Supplement: Supplementary file 1 — Additional file 1: Figure 1. [file 41016_2024_365_MOESM1_ESM.png]

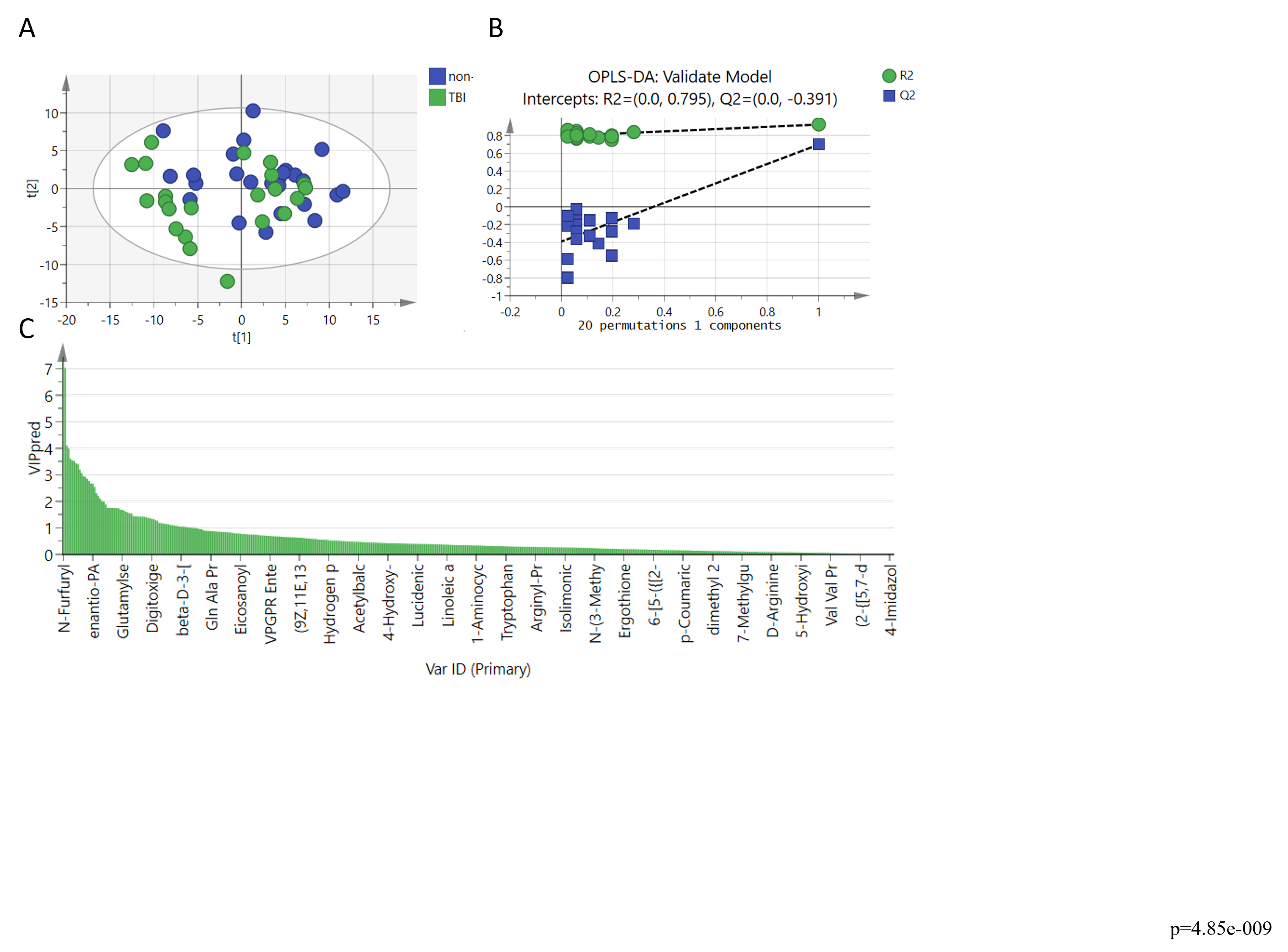

Supplement: Supplementary file 2 — Additional file 2: Figure 2. [file 41016_2024_365_MOESM2_ESM.png]

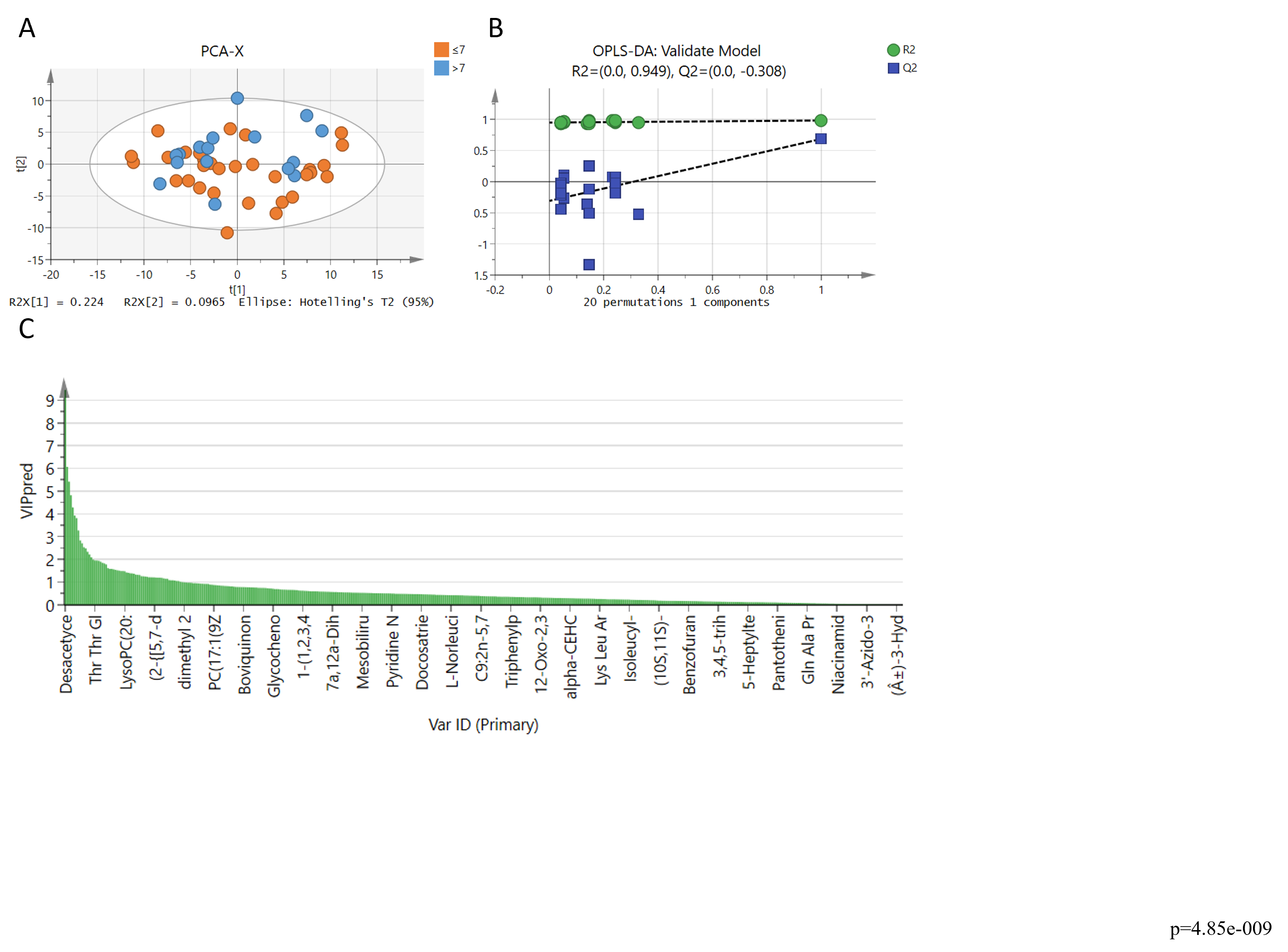

Supplement: Supplementary file 3 — Additional file 3: Figure 3. [file 41016_2024_365_MOESM3_ESM.png]

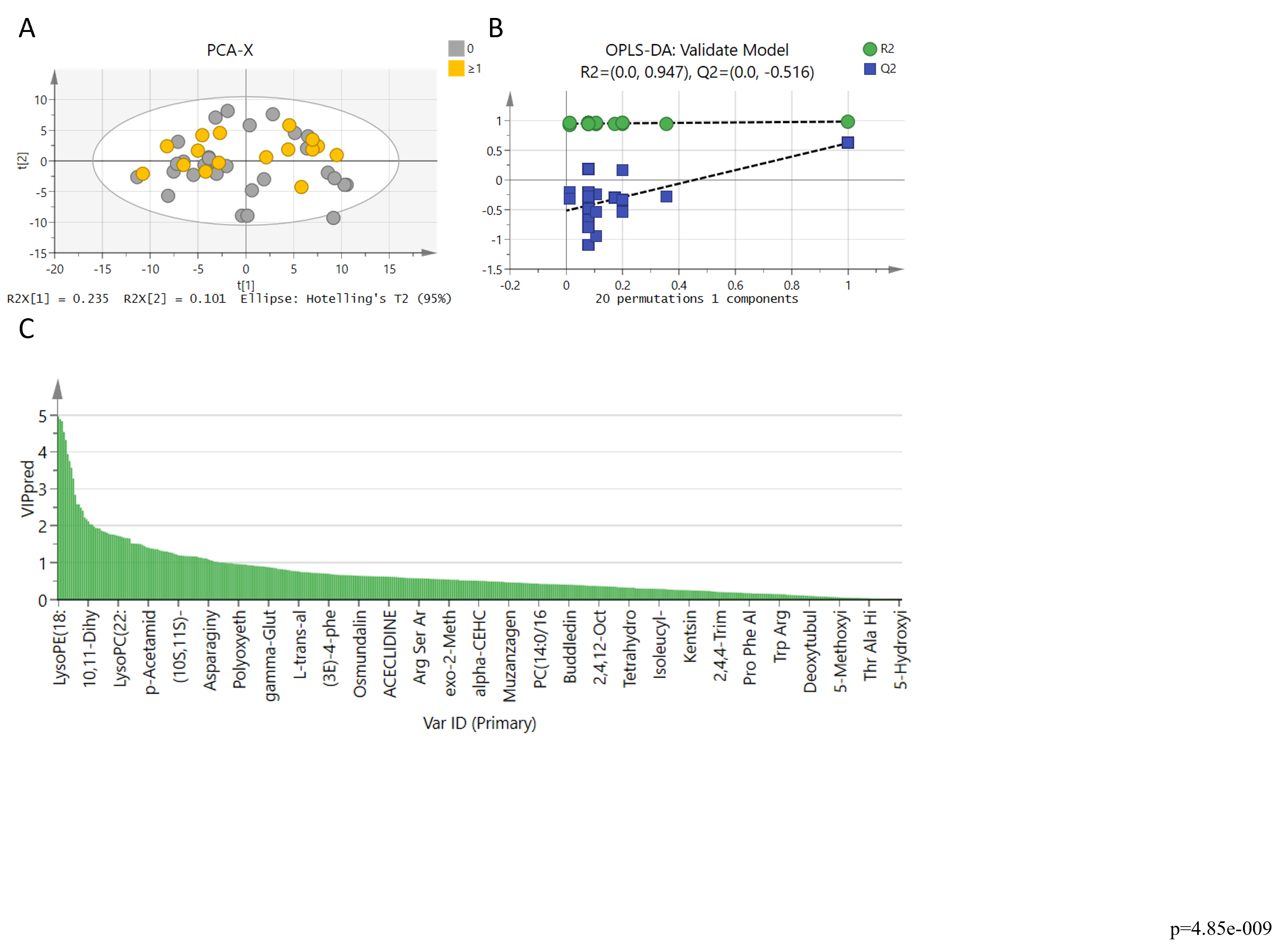

Supplement: Supplementary file 4 — Additional file 4: Figure 4. [file 41016_2024_365_MOESM4_ESM.png]
